# Case Report: Concurrent Occurrence of Abdominal Double Expressor Lymphoma and Jejunum Follicular Lymphoma

**DOI:** 10.3389/fonc.2021.656219

**Published:** 2021-05-26

**Authors:** Ryutaro Takada, Tomohiro Watanabe, Ikue Sekai, Keisuke Yoshikawa, Akane Hara, Yasuo Otsuka, Tomoe Yoshikawa, Ken Kamata, Kosuke Minaga, Yoriaki Komeda, Takaaki Chikugo, Yasuyuki Arai, Kohei Yamashita, Masatoshi Kudo

**Affiliations:** ^1^ Department of Gastroenterology and Hepatology, Kindai University Faculty of Medicine, Osaka-Sayama, Japan; ^2^ Department of Diagnostic Pathology, Kindai University Hospital, Osaka-Sayama, Japan; ^3^ Department of Hematology and Oncology, Kyoto University Graduate School of Medicine, Kyoto, Japan

**Keywords:** double expressor lymphoma, FISH, follicular lymphoma, transformation, MYC

## Abstract

Double expressor lymphoma (DEL), defined as overexpression of BCL2 and MYC, is an aggressive subtype of diffuse large B cell lymphoma (DLBCL). Here we report a case of a 64-year-old female diagnosed with abdominal DEL transformed from jejunum follicular lymphoma (FL). 18F-fluorodeoxyglucose (FDG)-positron emission tomography showed diffuse accumulation of FDG into the peritoneum and small bowel wall. Double balloon-assisted enteroscopy revealed whitish submucosal tumors in the proximal jejunum. Aggregation of atypical lymphocytes positive for CD20, CD79a, and BCL2 was seen in the jejunal biopsy samples. These atypical lymphocytes were monoclonal since cell surface expression of Ig light chains was limited to κ chain by flow-cytometry. Thus, immunohistochemical and flowcytometric analyses data were consistent with FL of the jejunum. Neoplastic lymphocytes obtained from ascites were positive for CD10, CD20, CD79a, BCL2, and BCL6. Fluorescence *in situ* hybridization (FISH) showed formation of *BCL2/IgH* fusion gene and extra copies of *MYC*, the former of which is a characteristic chromosomal abnormality of FL. These genetic alterations and protein expression profiles of ascitic fluid cells were consistent with those of DEL transformed from FL. Given that a significant population of patients with indolent FL of the gastrointestinal tract developed into aggressive DLBCL, it is likely that primary FL of the jejunum transformed into the abdominal aggressive DEL in this case. This case is unique in that concurrent occurrence of FL and DEL was confirmed by immunohistochemical and FISH analyses and that abdominal DEL transformed from jejunal FL was highly suspected.

## Introduction

It is now generally accepted that diffuse large B cell lymphoma (DLBCL) is a heterogeneous disease entity including a wide variety of pathologic findings and gene expression profiles (1, 2). Standard chemoimmunotherapy composed of rituximab, cyclophosphamide, doxorubicin, vincristine, and prednisolone (R-CHOP) results in remission in around 70% of patients with DLBCL (3). Recent progress in the understating of genomic alterations and oncogenic pathways enabled us to identify subtypes of DLBCL exhibiting aggressive clinical manifestations and poor outcomes. Double hit lymphoma (DHL) and double expressor lymphoma (DEL) are being recognized as such aggressive types of DLBCL. DHL is characterized by a dual rearrangement of *MYC* and *BCL2* and/or *BCL6* whereas DEL is defined as overexpression of MYC and BCL2 (4–6). Thus, it is very important to consider a possibility of DHL or DEL upon encounter with a case highly suspected aggressive DLBCL. In addition, cases with DHL or DEL transformed from indolent follicular lymphoma (FL) have been reported (6, 7).

Although DLBCL and FL frequently occur in the abdominal organs including the gastrointestinal (GI) tract, clinical manifestations of DHL or DEL arising from the abdomen have been poorly understood. Here we report a case with aggressive abdominal DEL, which was likely to be transformed from FL of the jejunum.

## Case Presentation

A 64-year-old female was admitted to our hospital due to abdominal distention. She had been treated for hyperlipidemia. On admission, she developed a low-grade fever and her superficial lymph nodes were not palpable. Physical examinations revealed abdominal distention caused by ascites. Leukocytosis (10,430/μL) and thrombocytosis (45.6x10^4^/μL) were seen in the context of complete blood cell counts. Biochemical analyses showed hypoproteinemia (Total protein 6.5 g/dL, normal range 6.6-8.1) and hypoalbuminemia (3.6 g/dL, 4.1-5.1). Although her serum concentrations of transaminases, blood urea nitrogen, and creatinine were normal, marked elevations of serum lactate dehydrogenase (LDH, 4,608 U/L, 124-222) and C-reactive protein (CRP, 2.196 mg/dL, <0.14) were noted. Tumor markers including carcinoembryonic antigen, carbohydrate antigen 19-9, alpha fetoprotein, and cancer antigen 125 were within normal range except for soluble IL-2 receptor (sIL-2R, 1,151 U/mL, 121-613).

**Figure 1:**
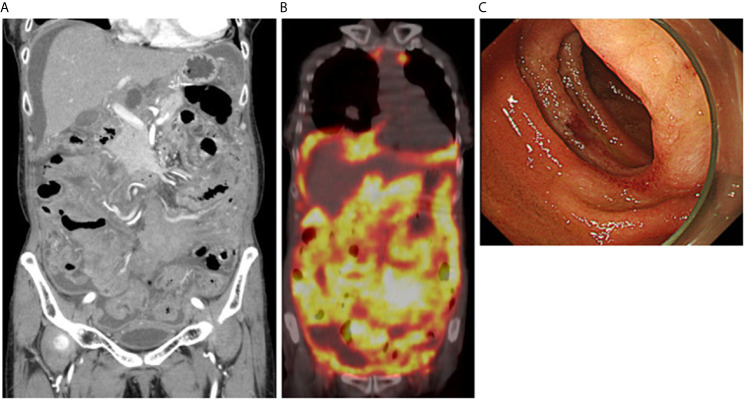
Massive accumulation of 18F-fluorodeoxyglucose into the abdominal cavity in positron emission tomography. **(A)** Moderate ascites and wall thickening of the jejunum and ileum were seen in contrast-enhanced computed tomography. **(B)** Massive accumulation of 18F-fluorodeoxyglucose was observed into the peritoneum and small bowel wall. **(C)** An endoscopic image of a jejunum tumor detected by double balloon-assisted enteroscopy. A whitish elevated tumor was seen in the proximal jejunum.

**Figure 2:**
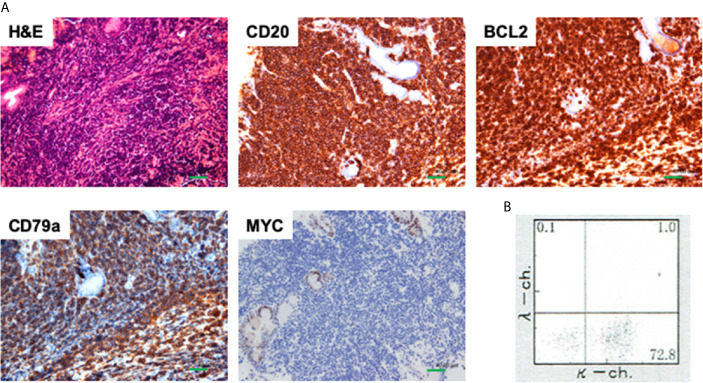
Pathological findings of jejunal biopsy samples. **(A)** Jejunal biopsy samples obtained from a whitish submucosal tumor were subjected to hematoxylin and eosin (H&E) staining and immunohistochemical analyses. Atypical lymphocytes were positive for CD20, and CD79a, and BCL2 whereas those were negative for MYC. Scale bar, 40 μm. **(B)** Cell surface expression of Ig light chains was limited to κ chain by flow-cytometry.

Moderate ascites and wall thickening of the jejunum and ileum were detected in abdominal computed tomography (CT, **Figure 1A**). 18F-fluorodeoxyglucose-positron emission tomography (FDG-PET) showed remarkable and diffuse accumulation of FDG (SUV max 12.5) into the peritoneum and small bowel wall (**Figure 1B**). No major abnormalities were seen esophagogastroduodenoscopy or colonoscopy. Capsule endoscopy was performed to explore the causes of marked wall thickening of the small bowel. A whitish elevated lesion with surface erosions and ulcers was detected in the proximal jejunum in capsule endoscopy (data not shown). Aggressive malignant lymphoma arising from the jejunum was strongly suspected in this case based on the presence of the jejunum tumor, remarkable uptake of FDG into the peritoneal cavity, and elevated serum levels of LDH and sIL-2R. Double balloon-assisted enteroscopy (DBE) was performed to verify this diagnosis. DBE detected a whitish elevated lesion with surface erosions in the proximal jejunum (**Figure 1C**). Written informed consent was obtained from this patient. Pathological examination using jejunum biopsy samples revealed infiltration of atypical small lymphocytes positive for CD20, CD79a and BCL2 and negative for MYC in immuno-histochemical (IHC) analyses, suggesting infiltration of atypical B cells in the jejunum (**Figure 2A**). These atypical lymphocytes were monoclonal since cell surface expression of Ig light chains was limited to κ chain by flow-cytometry (**Figure 2B**). Moreover, Ig heavy chain rearrangement was confirmed by polymerase chain reaction. Based on these data obtained by IHC and flow-cytometric analyses, jejunal tumor was diagnosed as FL (8, 9).

**Figure 3:**
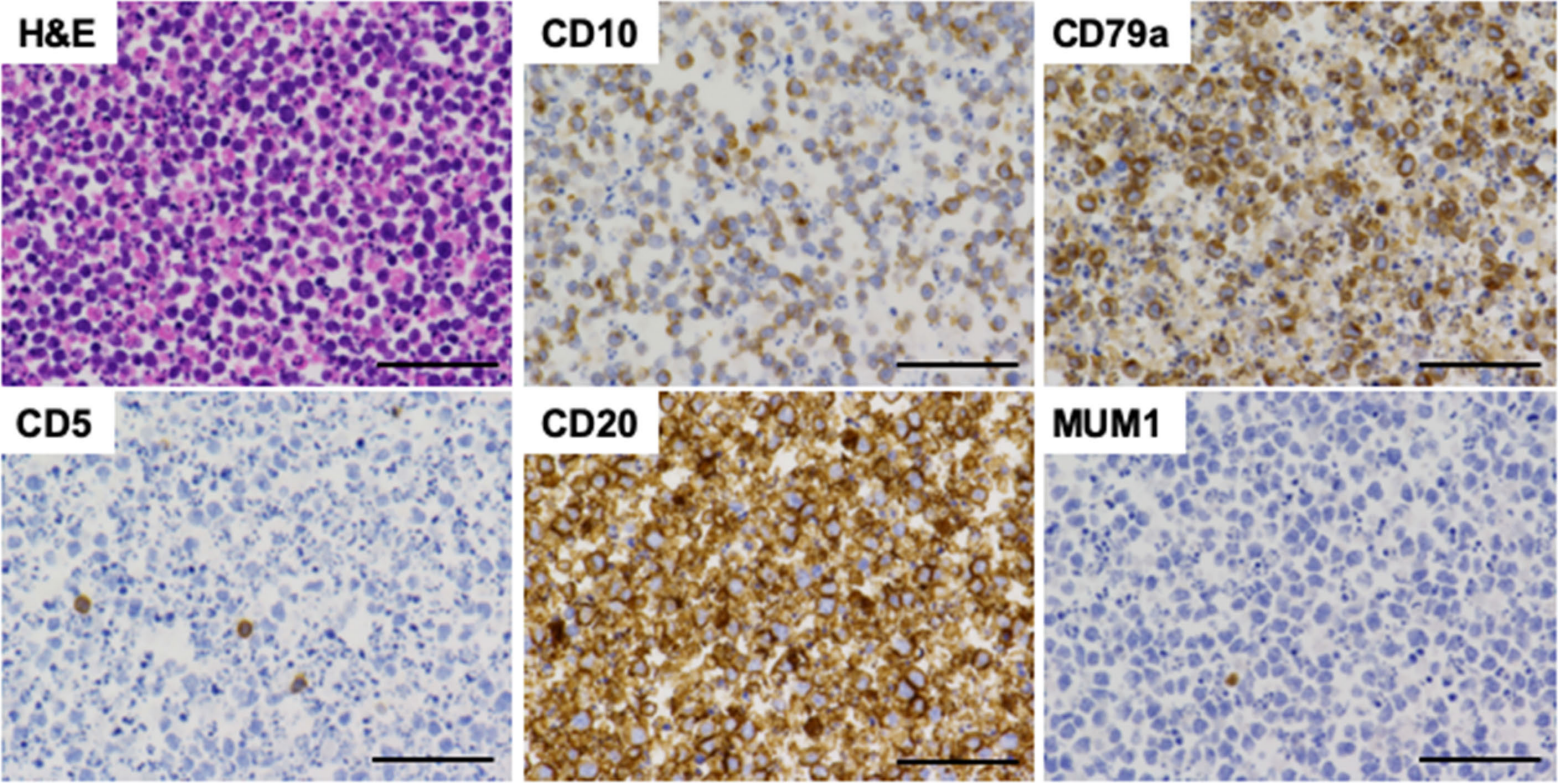
Pathological findings of ascitic fluid cell block samples. Ascitic fluid cell block samples were subjected to hematoxylin and eosin (H&E) staining and immunohistochemical analyses. Neoplastic lymphocytes were positive for CD10, CD20, and CD79a whereas those were negative for CD5 and MUM1. Scale bar, 50 μm.

**Figure 4:**
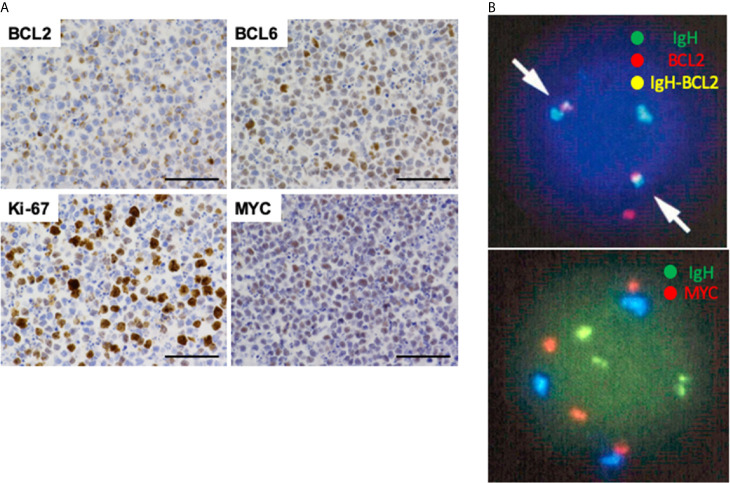
Immunohistochemical analyses and fluorescence *in situ* hybridization of ascitic fluid cell block samples. **(A)** Neoplastic lymphocytes were positive for BCL2, BCL6, and MYC. The percentage of Ki-67-positive cells was 70%. Scale bar, 50 μm. **(B)** Ascitic fluid cells were subjected to fluorescence *in situ* hybridization (FISH). FISH detected formation of *BCL2/IgH* fusion signal (arrows, yellow color) in 99 cells among analyzed 100 cells (top). Gain of *MYC* signal (red color) was seen in 97 cells among analyzed 100 cells (bottom).

Ascitic cell block specimens, which were prepared to verify the diagnosis of malignant lymphoma, were subjected to hematoxylin and eosin (H&E) staining and IHC analyses as previously described (4). H&E staining using ascitic fluid cell block showed aggregation of neoplastic lymphocytes (**Figure 3**). These neoplastic lymphocytes were negative for CD5 and MUM1 and positive for CD10, CD20, CD79a, BCL2 and BCL6 in IHC analyses (**Figures 3** and **4**). Although these IHC results were consistent with FL arising from the small intestine, aggressive phenotype seen in this case was atypical for FL. In fact, the percentage of neoplastic lymphocytes positive for Ki-67 was 70% (**Figure 4A**). DHL and DEL are being recognized as subtypes of DLBCL exhibiting aggressive phenotypes (4–6). DHL, defined as a dual rearrangement of *MYC* and *BCL2* and/or *BCL6*, shows poor outcome. In parallel to DHL, DEL defined as overexpression of MYC and BCL2 exhibits aggressive phenotype with poor prognosis (4–6). In addition, a case of FL transformed into DHL or DEL was reported (6, 7). To ascertain transformation of FL into DHL or DEL, fluorescence *in situ* hybridization (FISH) was performed. FISH showed *BCL2/IgH* fusion signal in 99 cells among total 100 cells examined (**Figure 4B**). Extra copies of *MYC* (**Figure 4B**) were observed in 97 cells among total 100 cells examined (**Figure 4B**), suggesting that neoplastic lymphocytes were characterized by over-expression of BCL2 and MYC. In line with FISH results, overexpression of MYC was seen in IHC analyses (**Figure 4**). Given that formation of *BCL2/IgH* fusion gene is one of the most characteristic chromosomal abnormalities in FL (8, 9), these FISH and IHC results strongly suggest that FL arising from the jejunum transformed into DEL and thus exhibited an aggressive phenotype characterized by massive accumulation of FDG into the peritoneal cavity. The cut-off values of the percentages positive for MYC and BCL2 in IHC analyses for the diagnosis of DHL are usually set as more than 40% and 50%, respectively (10). In our IHC analyses, those positive for MYC and BCL2 were around 40% and 30%, respectively, suggesting that expression of MYC and BCL2 in ascitic fluid cells might be weak for the definitive diagnosis of DEL. However, almost all of the ascitic fluid cells were positive for extra copies of *MYC* in FISH analyses. Therefore, FISH results strongly suggest the diagnosis of abdominal DEL.

Although DEL is resistant to R-CHOP, she was initially treated with R-CHOP due to very bad performance status (11). After one course of R-CHOP followed by six courses of chemoimmunotherapy composed of rituximab, gemcitabine, cyclophosphamide, vincristine, and prednisolone (R-GCVP), her ascites and bowel wall thickening were markedly decreased. Thus, chemotherapy was effective in this case with abdominal DEL.

## Discussion

Here we report a case with abdominal DEL, which was likely to be transformed from FL. FDG-PET showed massive accumulation of FDG into the peritoneum and small bowel wall, which findings together with elevated levels of serum LDH and sIL-2R were consistent with the diagnosis of aggressive type malignant lymphoma. The final diagnosis of DEL transformed from FL was done based on the results of IHC and FISH data. Neoplastic lymphocytes obtained from ascites were negative for CD5 and MUM1 and positive for CD10, CD20, CD79a, BCL2, and BCL6 in IHC analyses. Moreover, *BCL2/IgH* fusion signal, one of the most characteristic chromosomal abnormalities in FL, was detected in 99% of analyzed cells (8, 9). Although these IHC and FISH data were consistent with FL (8, 9), aggressive clinical course did not fit the diagnosis of FL. In fact, the fraction of Ki-67-positive tumor cells was 70%, suggesting highly proliferative lymphoma. We then considered the possibility of DLBCL transformed from FL by focusing on activation of MYC since MYC expression gain is a typical feature associated with indolent FL transforming to aggressive lymphomas (5, 6, 12). FISH and IHC analyses showed gain of *MYC* signals and overexpression of MYC protein, respectively. Therefore, neoplastic lymphocytes in this case were characterized by overexpression of BCL2 and MYC as well as formation of *BCL2/IgH* fusion gene. These FISH and IHC data fully support the diagnosis of abdominal DEL transformed from FL (5, 6, 10). The jejunal tumor was diagnosed with FL characterized by expression of CD20, CD79a, and BCL2. Concurrent occurrence of abdominal DEL and jejunal FL fully support the idea that jejunal FL transformed into the aggressive DLBCL. Although abdominal DHL transformed from inguinal lymph-node FL has been reported (6), co-occurrence of DEL and FL in the abdomen has not been reported.

DEL and DHL are subtypes of DLBCL with poor outcomes and aggressive clinical manifestations (5, 6, 10). DHL, defined as dual rearrangement of *MYC* and *BCL2* and/or *BCL6*, is considered a clinically aggressive neoplasma with a poor prognosis (5, 6, 10). In parallel to DHL, DEL, defined as overexpression of MYC and BCL2 in the absence of chromosomal rearrangements, shows an aggressive phenotype with poor outcomes (5, 6, 10). Given that DHL and DEL account for 5% and 20% of all DLBCL, respectively, we need to bear in mind a possibility of these aggressive subtypes upon encounter with a case of aggressive malignant lymphoma. Interestingly, Magnoli et al. reported that GI tract DLBCLs displayed a higher rate of gene rearrangements involving *MYC* as compared with DLBCLs of other sites including cervico-cephalic region, central nervous system, testes, and skin (13). Therefore, it is not surprising that DEL or DHL develops in the GI tract through activation of MYC. However, clinical manifestations of DEL or DHL arising from the abdominal organs including the GI tract have not been established yet. Massive uptake of FDG into the peritoneum and small bowel walls as well as diffuse wall thickening of small bowel may be one of characteristic findings of abdominal DEL.

Extranodal FL preferentially occurs in the GI tract (9). The GI tract FL most frequently occurs in the duodenum followed by the jejunum and ileum (9). Watch and wait strategy is recommended in most cases with GI tract FL due to clinical indolent behaviors and a very low incidence of transformation to DLBCL. On the contrary, recent retrospective analysis by Saburi et al. showed that histological transformation to DLBCL was observed in two cases (8.7%) among total 23 cases with duodenal FL (14). Thus, occurrence of histological transformation to DLBCL is not so rare and careful follow-up is absolutely required even for cases with indolent FL of the GI tract. This idea is fully supported by the clinical course of this case exhibiting the development of DEL, which was likely to be transformed from FL of the jejunum.

One question arising from this case is where FL transformed into DEL. In this regards, capsule endoscopy and DBE detected a whitish submucosal tumor in the proximal jejunum, which finding was consistent with those of malignant lymphoma of the GI tract (9). Accumulation of monoclonal κ chain^+^ lymphocytes positive for CD20, CD79a and BCL2 into the jejunum mucosa was seen. Given that CD20, CD79a and BCL2 are the prototypical markers for FL, these data on IHC and flow-cytometric analyses strongly support the diagnosis of FL of the jejunum. Therefore, we speculate that FL arising from the jejunum might have transformed into DEL. However, we could not perform IGHV sequencing to demonstrate that DEL and FL arose from the same clone due to limited amount of jejunal biopsy samples. Moreover, we could not confirm the presence of *BCL2/IgH* fusion gene by FISH to verify the diagnosis of FL. Therefore, further molecular evidence obtained by IGHV sequencing and FISH is required to verify that jejunum FL transformed into abdominal DEL in this case. Although less likely, we could not completely exclude the possibility that nodal FL arising from abdominal lymph nodes transformed into DEL in this case.

We report a case with abdominal DEL transformed from FL of the jejunum. Our case was unique in that concurrent occurrence of FL and DEL was confirmed by IHC and FISH analyses and that abdominal DEL transformed from jejunal FL was highly suspected. We need to bear in mind a possibility of DHL or DEL upon encounter with a case highly suggestive of aggressive malignant lymphoma even in the presence of an indolent type of GI FL. In such cases, FISH and IHC analyses might be very useful to confirm a dual rearrangement of *MYC* and *BCL2* and/or *BCL6* and overexpression of these proteins.

## Data Availability Statement

The original contributions presented in the study are included in the article/supplementary material. Further inquiries can be directed to the corresponding author.

## Ethics Statement

All procedures followed have been performed in accordance with the ethical standards laid down in the 1964 Declaration of Helsinki and its later amendments. Written informed consent was obtained from the patient for publication of this report.

## Author Contributions

RT, IS, KYo, AH, YO, and YK took care of the patient. RT and TW wrote the manuscript draft. TY, KK, KM and MK edited and revised the manuscript. YA and KYa analyzed chromosome and pathological data. TC performed pathological analyses. All authors contributed to the article and approved the submitted version.

## Funding

This work was supported in part by a Grant from Yakult BioScience Foundation.

## Conflict of Interest

The authors declare that the research was conducted in the absence of any commercial or financial relationships that could be construed as a potential conflict of interest.
